# Chromosome replication, cell growth, division and shape: a personal perspective

**DOI:** 10.3389/fmicb.2015.00756

**Published:** 2015-08-03

**Authors:** Arieh Zaritsky, Conrad L. Woldringh

**Affiliations:** ^1^Faculty of Natural Sciences, Ben-Gurion University of the Negev, Be’er-Sheva, Israel; ^2^Swammerdam Institute for Life Sciences, University of Amsterdam, Amsterdam, Netherlands

**Keywords:** bacterial cell division cycle, nucleoid complexity and segregation, size and shape determination, transertion, peptidoglycan biosynthesis

## Abstract

The origins of Molecular Biology and Bacterial Physiology are reviewed, from our personal standpoints, emphasizing the coupling between bacterial growth, chromosome replication and cell division, dimensions and shape. Current knowledge is discussed with historical perspective, summarizing past and present achievements and enlightening ideas for future studies. An interactive simulation program of the bacterial cell division cycle (BCD), described as “The Central Dogma in Bacteriology,” is briefly represented. The coupled process of transcription/translation of genes encoding membrane proteins and insertion into the membrane (so-called transertion) is invoked as the functional relationship between the only two unique macromolecules in the cell, DNA and peptidoglycan embodying the nucleoid and the sacculus respectively. We envision that the total amount of DNA associated with the replication terminus, so called “nucleoid complexity,” is directly related to cell size and shape through the transertion process. Accordingly, the primary signal for cell division transmitted by DNA dynamics (replication, transcription and segregation) to the peptidoglycan biosynthetic machinery is of a physico-chemical nature, e.g., stress in the plasma membrane, relieving nucleoid occlusion in the cell’s center hence enabling the divisome to assemble and function between segregated daughter nucleoids.

## Bacteriology and the Molecular Biology Revolution

Bacteriology was conceived by the Dutch Scientist Antony van Leeuwenhoek in the 17th Century (Porter, 1976), but considered “The Last Stronghold of Lamarckism” until 1943, when the ingenious Fluctuation Test was performed (Luria and Delbrück, 1943). The Phage Group of reductionists led by Max Delbrück (Cairns et al., 1966) revolutionized Basic Genetics to explain the flow of genetic information from Mendelian genes to proteins in molecular terms. This transformation was preceded by the era of protein biochemistry that could not easily pass the concept hurdle of enzyme-cannot-make-enzyme paradox (Stent and Calendar, 1978). Pure logic supported by simple, clear-cut experiments forced them to conclude that the long, seemingly monotonous DNA macromolecule is the storehouse of genetic information.

Molecular Biology developed quickly by clarifying that the transforming principle (Avery et al., 1944) was DNA: its structure was deciphered (Watson and Crick, 1953), semi-conservative replication demonstrated (Measelson and Stahl, 1958), functions in transcription/translation into proteins disclosed (Nirenberg, 2004), and manipulations crossed species barriers (Balbás et al., 1986). Two mutually-exclusive groups that hardly exchanged information were responsible for the revolution: those mentioned above led by Physicist Max Delbrück and Chemists led by Arthur Kornberg (Kornberg and Baker, 1992). Exposing the DNA’s symmetrical beauty and crucial role required seminal studies by persistent scientists such as Erwin Chargaff and Rosalind Franklin, who were in the frontier’s cutting edge but individualistic and less lucky (Watson, 1996).

There were unavoidable diversions: some excellent scientists considered penicillin resistance to be an adaptive response, others described enzyme induction in terms of kinetics, still others thought of bacterial conjugation as zygote formation, but these and many more failed attempts were indispensable for the main thrust of advance. Furthermore, the absolute acceptance of the operon model (Jacob and Monod, 1961) for example, misled others to consider *lon* mutants as regulatory (Markovitz, 1964).

Merging molecular biology with general bacteriology, basic genetics and sophisticated microscopic and physical techniques discovered the sexuality and circularity of the bacterial chromosome (Jacob and Wollman, 1956; Cairns, 1963; Hayes, 1968), its replication schedule (Helmstetter et al., 1968), and the nucleoid structure (Kellenberger et al., 1958; Woldringh and Odijk, 1999).

## The Origins of Bacterial Physiology

Until the late 1920’s, bacterial cultures were thought to be composed of cells that constantly change size, form and structure in a meaningless fashion. In his book, Henrici (1928) noted that these changes during a single growth cycle “*occur with great regularity and are governed by simple laws which,… may probably be very precisely formulated*.” It took 30 years to achieve this goal in descriptive terms, and additional decades to begin deciphering the fundamental laws anticipated by Henrici (1928) in robust, molecular terms. The multitude of forms and sizes in a random, single-species pure culture could only be explained when age distribution (Powell, 1956) and balanced growth (Campbell, 1957) were defined, and the Copenhagen School (Maalϕe and Kjeldgaard, 1966; Schaechter, 2006) described how cell size and composition change with the medium (Schaechter et al., 1958) and during transitions between growth rates (Kjeldgaard et al., 1958).

Ole Maalϕe was working at The State Serum Institute (Cooper, 1993) until he was named a Professor and started, late in 1958, The Institute of Microbiology. It seems to some of us that Ole entertained the idea to imitate Niels Bohr’s Physics Institute, likely because he held Bohr in the highest admiration and was a good friend of his son Aage, also a Nobel laureate in Physics. This Institute and Ole’s strong personality influenced dramatically several generations of scientists involved in investigating physiological aspects of the bacterial cell, nicknaming it The Copenhagen School. The numerous scientists who passed through it during their careers (Anderson et al., 2006), mostly young, promising and subsequently influential, demonstrate that it was a success.

The seminal series of experiments with *Salmonella typhimurium* published in 1958 in two back-to-back articles (Kjeldgaard et al., 1958; Schaechter et al., 1958), established the field of Bacterial Physiology and turned into its main hallmark. The stream of articles stemming from the Institute became a flood of crucial information published in the most prestigious periodicals of the time. One major motto of Ole in understanding the cell was “Look–Do Not Touch” hence studies were performed with minimal perturbations of the so-called steady-state of exponential growth (Fishov et al., 1995). After physiological manipulations were seemingly exhausted, the use of drugs and mutants became common when the mechanisms of their actions were, or thought to be deciphered. The multi-faceted phenotypes exerted by these (lack of specificity and pleiotropism, respectively) occasionally remind us to stick to this rule-of-thumb in order to keep interpretations of results as crystal-clear as possible.

This first leg of the journey to understand the logic behind the duplication of a bacterial cell, which took place in the 1950s, is described in this collection by Schaechter (2015), and the other two, partially overlapping legs in the 1960s–by Hanawalt (2015) and Helmstetter (2015). Phil studied the phenomenon of thymineless-death (TLD) in thymine-starved populations of *thyA* mutants (Cohen and Barner, 1954) employing it to better understand the connection between chromosome replication and cell growth and viability (Hanawalt et al., 1961), and Charles exploited the neat, so-called “baby-machine” that he devised (Helmstetter and Cummings, 1964) to derive the temporal aspects of the bacterial cell cycle (Helmstetter et al., 1968).

Being students during the early 1970’s, here we try to fill-in the development in a perspective of half a century and in line with our view-points. To this effect, we acknowledge with admiration the ingenuity of Noboru Sueoka and Hirosho Yoshikawa, whose results with *Bacillus subtilis* (Yoshikawa and Sueoka, 1963) revealed Ole’s prediction (Maalϕe, 1961) that replication initiates from a single point (later defined as *oriC*) and is sequential and multi-forked at fast growth rates (Oishi et al., 1964). Thinking rigorously, they derived marker frequency equations (Sueoka and Yoshikawa, 1965) that survived the test of time. Bidirectionality of the replication has later been demonstrated by various genetic, physiological and microscopic means (e.g., Masters and Broda, 1971; Bird et al., 1972; Prescott and Kuempel, 1972; Wake, 1972).

Experiments that investigated the fractional increase of DNA (Δ*G*) in amino acids-starved cultures of *Escherichia coli* 15T^–^ (so-called “runout”) using dense and radioactive isotopes of thymine (Lark et al., 1963) led to the discovery of the so-called premature initiation (Pritchard and Lark, 1964), distinguishing between the two independent processes of replication, initiation and elongation. This distinction had clearly been indicated by Phil’s classical experiments (Hanawalt et al., 1961), and was later supported by isolating two groups of conditional-lethal replication mutants (Hirota et al., 1968) that either stopped replication immediately upon transfer to the restrictive temperature (elongation) or allowed completion of the ongoing cycle but not new initiations.

## Growth, Chromosome Replication and Cell Division; the BCD

Two essential, unique macromolecules (structures) exist in a bacterium: DNA (nucleoid) that stores the genetic information, and the shape-maintaining peptidoglycan (sacculus), which also protects the cell from rupture by its osmotic pressure (turgor). To survive, the cell must divide after its genome doubles and in a plane between the two emerging sets, hence duplications of the two are coupled, temporally and spatially. Much effort is expended to discover the mechanism responsible for this coupling, which raises the efficacy of competition among species. To study this coupling, reproducible steady-state conditions and well-defined perturbations (Maalϕe and Kjeldgaard, 1966) have been exploited.

Wild-type *E. coli* can synthesize all of its component macromolecules necessary for duplication from aqueous salts solution. Multiplication rate is carbon source-dependent, the most efficient of which is glucose, supporting doubling time *τ* of about 40 min at 37°C. Slower rates are obtained on poorer sources, whereas adding organic building blocks result in faster rates, the maximum achievable being about 3 h^–1^ (i.e., *τ*_*min*_ ≈ 20 min). Irrespectively, the time *C* taken to duplicate the chromosome (of ∼4.6 Mb) is constant, *ca*. 40 min (Helmstetter et al., 1968). A cell divides into two morphologically-identical daughters (Trueba and Woldringh, 1980) about 20 min (designated *D*) after termination of replication hence division follows replication-initiation by about 1 h. This model was experimentally confirmed for cells growing at *τ* ranging 20–70 min (growth rate μ of 3–0.9 h^–1^, respectively). Situations with *τ* < *C* are achieved by initiating new replication rounds before completing the previous ones. Under slow growth rates, on the other hand, the cycle includes a period *B* [ = *τ* – (*C*+*D*)] in which cells have not initiated yet hence they continue to grow—much like in the G_1_ period of the eukaryotic cell division cycle. This (*B*, *C*, *D*, *τ*) model has survived over 40 years with minor modifications of parameter values (e.g., Bipatnath et al., 1998; Michelsen et al., 2003), and many of its conclusions have been confirmed in other eubacteria (Helmstetter, 1996; Toro and Shapiro, 2010). It can thus be termed (Zaritsky et al., 2011, 2012) as “*The Central Dogma of The Bacterial Cell Division Cycle*” (two meanings for *BCD*). A cell cycle is divided in 3 (or 4) periods by two major events between successive fissions, initiation and termination of replication that can occur in reverse order depending on the values of *C*, *D*, and *τ* (Jiménez Sánchez, 2015).

Combining the noted constancy of *C* and *D* values (Helmstetter et al., 1968) with the way mean cell mass change with *τ* (Schaechter et al., 1958) resulted in an important insight: cell mass *M*_*i*_ at the time of replication-initiation is roughly constant per replication origin *oriC* (Donachie, 1968; Pritchard, 1968; Pritchard et al., 1969). The molecular mechanism regulating initiation of replication, occurring synchronously from all existing *oriC* copies and once per cell cycle, is under investigation (e.g., Leonard and Grimwade, 2010), but the apparent constancy of the *Mi*/*oriC* ratio is very useful, conferring a quantitative description of the bacterial cell. The cycle ends *C*+*D* min after initiation, when cell mass reaches *M*_*i*_ × 2^(^*^C^*^+^*^D^*^)/^*^τ^*. The changing exponential rate of mass growth in different media is not matched by the linear, constant DNA elongation rate (1/*C*), but the faster increase of cell mass in richer media leads to increased initiation frequency as prescribed by the constant *M*_*i*_/*oriC*. BCD thus explains changes in cell composition and size with *τ* and predicts the consequences of perturbations such as nutritional shifts (Kjeldgaard et al., 1958). These basic features and other examples are illustrated and can be followed by the user-friendly Cell Cycle Simulation program (CCSim) at https://sils.fnwi.uva.nl/bcb/ that was partially described before (Zaritsky et al., 2006, 2007, 2011, 2012) and will be re-mentioned below. It must be noted that the values of these constants do change slightly with *τ*—more so at longer values, can be manipulated experimentally by various means (e.g., Meacock and Pritchard, 1975; Zaritsky and Zabrovitz, 1981; Wold et al., 1994; Bipatnath et al., 1998), and inserted in the CCSim program to confirm or reject working hypotheses.

## Dissociating Rates of Replication and Growth

Capitalizing on Helmstetter’s “baby machine” (Helmstetter and Cummings, 1964) and just before the description of BCD (Helmstetter et al., 1968), Clark and Maalϕe (1967) demonstrated a constant rate of replication along the chromosome, with distinct discontinuities in DNA synthesis rate during the cell cycle interpreted as occurring due to initiation and completion of replication cycles. Chromosomes with multiple replication forks (also termed dichotomously replicating) is the reason for bigger Δ*G* added DNA in amino acids-starved, faster growing cells (Schaechter, 1961). This was the current knowledge at the end of 1968, upon the arrival of one of us (AZ) at Leicester University for graduate studies, supervised by Robert Pritchard, who had established the Genetics Department there merely 4 years earlier^1^.

Digressing to some personal involvements, one of us (AZ) was very lucky to enter the atmosphere inspired by Bob and at the right time to be assigned a project in the just-opened BCD field, about which I had no clue. During 6-years of previous studies (1962–1968) at the Hebrew University of Jerusalem, my M.Sc. (with distinction but no publication) in Bacterial Genetics was supervised by Amiram Ronen, I finished 4 full years of Pre-Medical studies and attended several courses in Mathematics (my ever-lasting love). The latter was helpful to sharpen rigorous thinking, to derive the equation relating DNA concentration to the number of replication positions (Sueoka and Yoshikawa, 1965) *n* ( = *C*/*τ*) irrespective of the value of *D* (Pritchard and Zaritsky, 1970; Zaritsky, 1971) and to program the huge computer at Leicester University (using card-punching). It may have been important for my active participation in developing CCSim, as described below. Bob and his large team of students were instrumental for my learning both, proper English and the BCD, mainly in the tea/coffee/seminar room that was inhabited during many hours, days and nights.

Simultaneously, the other (CLW) extended his biological and microscopic skills at the University of Amsterdam. There are at least three at that time commonly-accepted ideas that I ruled out during my Ph.D. studies and beyond namely, existence of direct DNA-membrane attachments (Woldringh, 1974), of peri-septal annuli (Woldringh, 1994) and rapid nucleoid displacement (van Helvoort and Woldringh, 1994), all has meanwhile disappeared from our knowledge-base, justifiably so. My close association with Nanne Nanninga (e.g., Woldringh and Nanninga, 1985), who in the late 1960’s demonstrated the artifactual origin of mesosomes (Nanninga, 1971), enabled the establishment of a department that attracted distinguished students and scientists from all over the world, microbiologists as well as physicists and engineers. In their search to define the structural changes occurring during fixation and dehydration necessary for visualizing the bacterial nucleoid in the electron microscope, the possibilities to study live cells were improved with the reinvention and development of the confocal scanning light microscope (CSLM) by Brakenhoff (see Valkenburg et al., 1985).

Back to the main subject, at Leicester, Bob realized existence of literature-recorded contradictory results, the common feature of most is that they were obtained in thymine-requiring strains. These observations (e.g., Maalϕe and Rasmussen, 1963; Friesen and Maaloe, 1965; Lark and Lark, 1965; Beacham et al., 1968) led him to hypothesize that the replication time of the chromosome in *thyA* strains depends on the external concentration of thymine [T] present in their growth medium (Pritchard, 1974). This hypothesis could explain all discrepancies and is consistent with lack of active thymine-transport, in *E. coli* (Itsko and Schaaper, 2011) and other bacterial species (Carmody and Herriott, 1970; Reinhart and Copeland, 1973). It was strongly confirmed by four physiological methods, more or less independent of each other (Pritchard and Zaritsky, 1970; Zaritsky, 1971), and later supported by various means in other laboratories (reviewed in Zaritsky et al., 2006).

Thus, the dissociation between syntheses rates of mass and DNA, originally observed by changing the former alone (Helmstetter et al., 1968), was confirmed by exclusively manipulating *C* by limiting [T] in *thyA* strains (Pritchard and Zaritsky, 1970), affected through the intracellular [dTTP] (Beacham et al., 1971). This method is more amenable to analysis than nutritional shifts because modulating [dTTP] by changing [T] occurs abruptly, without affecting the multitude of metabolic pathways and interactions between them that accompany nutritional shifts (Scott and Hwa, 2011).

## Dissociating Cell Growth and Division; the Eclipse

In a steady-state exponentially growing culture, concentrations of all cell components increase in parallel to each other and in pace with divisions (Campbell, 1957; Fishov et al., 1995). The puzzling phenomenon of division rate-maintenance after a nutritional shift-up (Kjeldgaard et al., 1958) was instantly explained by the BCD model (Helmstetter et al., 1968): a cell divides a constant time, *C*+*D* min after initiation of chromosome replication, which in turn follows mass growth. The division-rate therefore changes *C*+*D* (*ca*. 65) min after the change in growth rate is affected by enriching the medium. Most perturbations, by chemical/physical agents or under restrictive conditions of *ts* mutants, cause immediate block of division (Slater and Schaechter, 1974)—one that is usually restored upon transfer back to permissive conditions. Specific inhibition of protein or DNA synthesis, however, allows divisions to continue during the *D* period; these so-called residual divisions cause a decrease in average cell length (cf. entry into stationary phase) and enable estimation of the *D* period (Dix and Helmstetter, 1973; Kubitschek, 1974; Woldringh et al., 1977).

Determination of *C* and *D* periods for batch cultures of *E. coli* cells have also been performed by flow cytometry (Michelsen et al., 2003) or by image cytometry (cf. Huls et al., 1999). From these studies it becomes clear how these cell cycle periods can vary with different strains and growth conditions. The measurements indicate that the *D* period is especially variable, making it difficult to generalize the *E. coli* cell cycle.

When thymine-limited *thyA* mutants grow at fast growth rates, another puzzling phenomenon appears, namely dissociation between growth and division that is related to replication. Under these conditions, the inter-division time is longer than mass doubling time (i.e., *τ*_*d*_ > *τ*_*m*_) thus cell size increases continuously (Zaritsky and Pritchard, 1973), and seemingly indefinitely. The 40 years-old observation (Zaritsky, 1975a) that indicated existence of a minimal possible distance *l*_*min*_ between two successive replisomes, promptly explains this phenomenon (Zaritsky et al., 2007). The question whether the mechanism involved is structural (replisome size; Norris et al., 2007) or chemical (sequestration of membrane-attached hemi-methylated DNA; Olsson et al., 2002) remains moot, but breaching this distance would extend the inter-initiation time *I* ( = *τ*_*i*_) beyond the mass doubling time (*τ*_*m*_) thus delay initiations, and cumulatively so (Zaritsky et al., 2007). Such a breach can be achieved by enhanced initiation frequency (Simmons et al., 2004) or slowed replication rate (Zaritsky and Pritchard, 1973). This distance is estimated to be about half of the chromosome length (*l*_*chr*_), termed the Eclipse (*l*_*min*_/*l*_*chr*_) and can be expressed in units of time depending on the rate of replication (*l*_*min*_/*l*_*chr*_)× *C* (e.g., how long it takes to reach this fraction of chromosome at a given, constant rate *C^–^*^1^). Release from this situation by restoring the permissive conditions causes a transient increase in the frequency of divisions (Zaritsky et al., 2011) thus substantiating this concept and facilitating its investigation.

## The Cell Cycle Simulation Program

**FIGURE 1:**
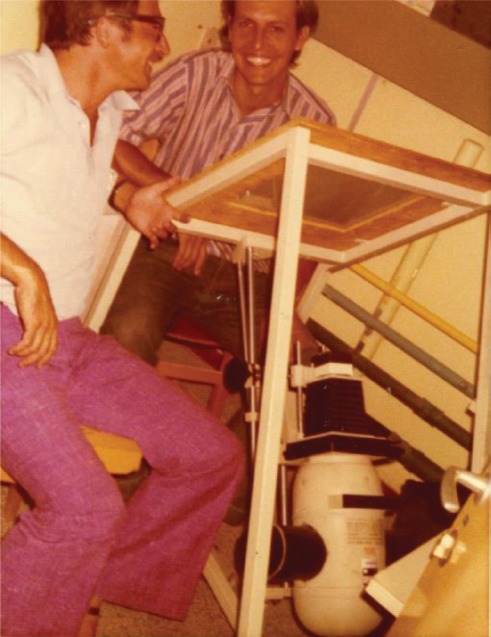
**Conrad (R) and Arieh (L) at the unique “Chezi” 30 mm-film projector, manually measuring cell dimensions and constrictions, Be’er-Sheva, Summer 1976.** This primitive, bulky “machine” was designed and constructed by the Workshop of the Natural Sciences Faculty at Ben-Gurion University, led by Mr. Yechezkel Tahori in the “pre-history” of computer visualization, initiated in the University of Amsterdam by (Trueba and Woldringh, 1980) and developed further into a versatile measuring plugin “ObjectL,” which runs under ImageJ (see Vischer et al., 2015).

Our fortuitous encounter at the Luntern Conference in November 1974 was very fortunate. We had apparently met 3 years earlier in a previous meeting there, but being students it hadn’t engendered significant mutual impressions. In 1974, both of us had already acquired results related to morphometric variations of *E. coli* cells under different growth conditions, theoretical (Zaritsky, 1975b) and experimental (Woldringh, 1974), and ideas about joint research sprang in the air during a long night of extensive discussions. It was just 7 months later that EMBO financed a 3-month visit for CLW in Be’er-Sheva (Figure 1), followed by another short-term fellowship for AZ to visit Amsterdam a couple of years later. These and follow-up visits culminated in detailed descriptions of cell dimensional rearrangements during nutritional shift-up experiments (Grover et al., 1980; Woldringh et al., 1980; Zaritsky et al., 1982), organization of two EMBO Workshops on Duplication of Bacteria (1980 in Holland; 1984 in Israel^2^), and 40 years of continuous cooperation. One notable outcome of our interactions was implementation of an interactive simulation program (Zaritsky et al., 2011) that integrates all quantitative knowledge about the BCD (Helmstetter et al., 1968), including the anticipated behavior of various existing and prospective mutants. This program implementation was enabled by the recruitment of Norbert Vischer, a computer engineer, by the Amsterdam department chair and faculty dean Nanne Nanninga. The lab in Swammerdam Institute is thus frequently referred to as The Amsterdam School (*à la* the Copenhagen School mentioned above).

**FIGURE 2:**
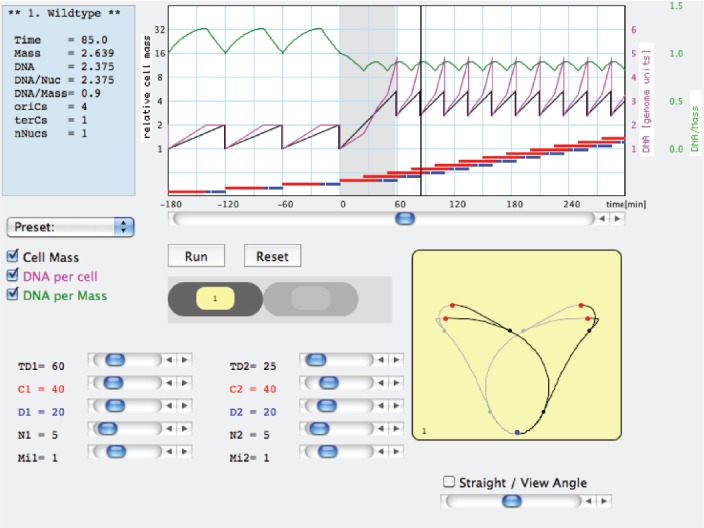
**Example of a work window of the Cell Cycle Simulation program (CCSim) for a nutritional shift-up from a doubling time τ_1_ = 60 min to τ_2_ = 25 min.** The interactive program can be downloaded from: https://sils.fnwi.uva.nl/bcb/.

All considerations described so far and by the CCSim (Figure 2) do not relate to cell dimensions and shape nor to nucleoid segregation. Future versions of CCSim may be extended to incorporate these aspects.

## Cell Size and Dimensions

An exponentially growing bacillary cell elongates with unnoticeable change in width, and divides evenly at a perpendicular plane (Trueba and Woldringh, 1980). The seminal observation (Schaechter et al., 1958) that larger cells at faster growth rates in richer media are both longer *and* wider led to the proposal (Zaritsky and Pritchard, 1973; Pritchard, 1974; Zaritsky, 1975b) that cell dimensions and cell shape could be directly coupled to the process of DNA replication and segregation. It was initially interpreted to involve active regulation of length *L* (Grover et al., 1977) or surface area *S* (Rosenberger et al., 1978a,b) extension, and passive response of width *W* to the changes of volume *V* and *L* (or *S*), the so-called linear/log model. Cell elongation was assumed to proceed at a constant rate (either dependent on μ or not) that is proportional to the number of *oriC*s, *terC*s (replication termini) or replisomes (Zaritsky and Pritchard, 1973). This view was later abandoned when peptidoglycan synthesis was demonstrated to be diffuse throughout the cylindrical periphery and only localized during the division process (Woldringh et al., 1987).

**FIGURE 3:**
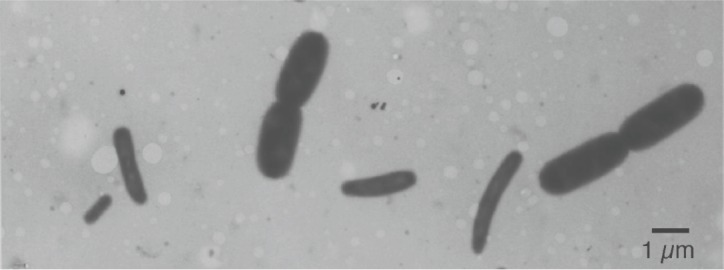
**Electron micrograph of a mixture of two ***E. coli*** B/r cultures prepared by agar filtration.** The big cells were grown in trypton broth with a doubling time of 22 min; the small cells were grown in synthetic alanine-medium with a doubling time of 150 min. Compare with a similar preparation of mixed populations in Figure 2 of Nanninga and Woldringh (1985).

**FIGURE 4:**
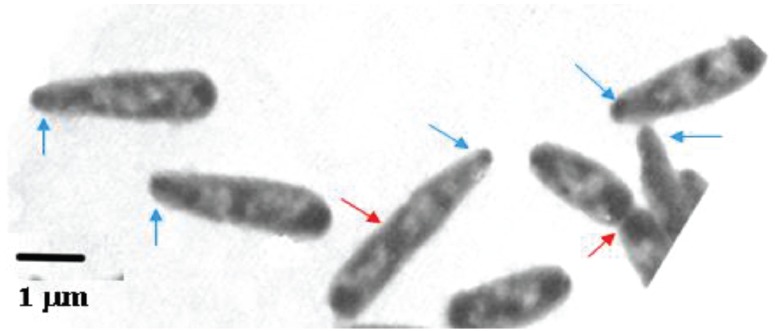
*****Escherichia coli*** B/r cells prepared for electron microscopy by agar filtration, 60 min after a nutritional shift-up from *τ*_1_ = 72 to *τ*_2_ = 24 min (cf. Figure 3 in Nanninga and Woldringh, 1985).** The nucleoids show up as electron-transparent regions in the air-dried cells, flattened by surface tension (cf. Woldringh et al., 1977). Red arrows indicate constriction sites, blue arrows, tapered tips.

With such models in mind, we measured (Figure 1) the dimensions of *E. coli* cells cultured under steady-state of exponential growth in different media supporting various rates, prepared for electron microscopy by the agar filtration method (Woldringh et al., 1977; Figure 3), and compared the results with the various models (Zaritsky et al., 1982). Our nutritional-upshift experiment (Woldringh et al., 1980) revealed that the increase in cell diameter was slow and occurred mainly during the division process in the vicinity of the deepening constriction site, forming transiently tapered cells (Figure 4). Consequent to this slow adaptation and almost immediate change in the rate of mass synthesis, cell length overshoots, but the mechanism governing this diameter change is still enigmatic. A diameter increase during the constriction process has also been implied in populations growing in steady state where the cells showed a diameter decrease during elongation (see Figure 4 in Trueba and Woldringh, 1980). It should be noted that in all these preparations the cells had been fixed with osmium tetroxide and were air-dried, causing their flattening (Vardi and Grover, 1993). Nevertheless, the measurements compared well with those obtained from hydrated cells with phase-contrast microscopy (cf. Table 3 in Trueba and Woldringh, 1980).

Associated with cell widening, the nucleoids (bright areas in Figure 4) start replicating in planes tilted to the long cell axis (Figure 4), rather than parallel to it as during slow growth conditions. The differences in cell dimensions and nucleoids replication-planes are pronounced when *thyA* cells grow under identical conditions but with limiting [T] that impose slow replication rate (compare, e.g., panels A and B of Figure 6 of Zaritsky et al., 2006; and see Figure 1 in Woldringh et al., 1994).

## Homeostasis of Cell Size and Shape

**FIGURE 5:**
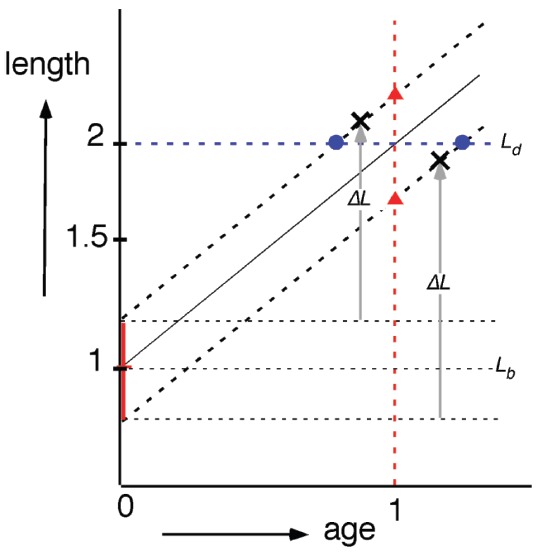
**Semi-log plot of cell length as a function of cell age (cf. Figure 6 in Koppes et al., 1978).** Irrespective of newborn cell size (vertical, red bar; *L*_*b*_ ± SD) cells elongate exponentially (same specific elongation rate). According to the “timer” model (red triangles), newborn cells divide (at age 1) after a constant period; according to the “sizer” model (blue circles, *L*_*d*_), newborn cells divide after reaching a critical size (at length 2); according to the “adder” model (black crosses) newborn cells divide after elongating with a constant length increment Δ*L*.

In the 1970’s, the period of DNA replication during a division cycle was determined by pulse-labeling cells with ^3^[H]-thymidine and measuring size distributions of cells prepared for radio-autographic electron microscopy (Koppes et al., 1978). These studies clarified that individual cells elongate exponentially (i.e., at a rate proportional to their length) and provided information about length variations at different events in the cycle as well as size and time correlations between these events (Koppes and Nanninga, 1980). The results led Koppes et al. (1978) in The Amsterdam School to propose that cells initiate constriction after a constant length increment Δ*L* following initiation of DNA replication (Figure 5) thus establishing a correlation between cell sizes at replication initiation and at initiation of visible constriction *C* min later. This model of constant Δ*L* was recently revived (Amir, 2014) and supported by measurements of live cells (Campos et al., 2014; Iyer-Biswas et al., 2014; Taheri-Araghi et al., 2015) confirming that a growing bacterium maintains stable size by adding a constant incremental length Δ*L* each generation irrespective of its size at birth. This automatically leads to size homeostasis that is valid at all growth rates obtained in different media, and since faster growing cells are longer, Δ*L* changes accordingly.

Jun and colleagues (Jun and Taheri-Araghi, 2014; Taheri-Araghi et al., 2015) proposed that the molecular mechanism underlying the size homeostasis by the so-called “adder” model (Figure 5) is related to the P-sector proteins of the *E. coli* proteome of which the total number per cell is relatively constant at different growth conditions. According to this hypothesis, accumulation of these proteins to a fixed threshold each generation would serve as a trigger for cell division. This proposal, however, does not relate mass growth to the DNA replication cycle, as suggested four decades ago (Zaritsky, 1975b). If P-sector proteins are at a fixed number per cell, then they would become diluted during the interdivision time (molecules fixed, but cell volume increases). Therefore, it is not clear how it could result in their accumulation to trigger division. Other aspects of this idea have recently been rebutted in more details (Zaritsky, 2015).

Coupling between DNA replication and cell elongation could be obtained by the nucleoid occlusion mechanism that is being relieved when daughter nucleoids are segregating apart (Mulder and Woldringh, 1989; Nanninga et al., 1990; Woldringh et al., 1990). This would require that newborn cells contain nucleoids with the same amount of DNA (*G/terC*) irrespective of their size at birth and that the state of nucleoid segregation parallels the cell’s length increase. In other words, a length increment of the nucleoid would be sensed rather than a length increment of the cell. That DNA replication and segregation go hand in hand with cell elongation is supported by observations on the movement of duplicated *oriC*’*s* (Elmore et al., 2005) and of segregating chromosome arms (cf. Youngren et al., 2014; Woldringh et al., 2015). However, while during slow growth all newborn cells can be assumed to contain nucleoids with the same amount of DNA, this will not hold for fast growth showing multifork replication. Here, stochastic premature or postponed division of mother cells will produce small and large daughter cells, respectively, with different amounts of DNA per nucleoid and thus different stages of segregation. Such cells will not signal division after a constant length increment as predicted by the “adder” model.

Another proposal (Ho and Amir, 2015; see also Robert, 2015) couples DNA replication and cell elongation to the time of initiation of DNA replication. Here, sensing of a constant length increment is starting at the last initiation of DNA replication. How a size increment rather than a critical size is monitored and whether nucleoid segregation is involved in such a model remains to be seen. Presently, information is lacking on the size of the nucleoids in newborn cells at different growth rates at the individual cell level. Better DNA staining techniques are required to observe nucleoid growth and segregation in individual cells growing in microfluidic systems.

Whatever property a cell is sensing to enable it to divide after a constant size increment irrespective of its size at birth, some communication will be necessary between the dynamics of DNA (transcription, replication and segregation) and the biosynthetic activities of peptidoglycan (elongation and constriction at perpendicular angles). It has been proposed (Rabinovitch et al., 2003) that DNA could exert stress on the membrane through the transertion mechanism (Woldringh, 2002): coupled transcription/translation of genes encoding membrane proteins and inserting these proteins into the membrane. The strength of this interaction varies along cell length with a minimum in between the segregating nucleoids. By a yet-unknown mechanism, this stress-change signal that is relayed to initiate division is proposed to be sensed by the peptidoglycan-synthetic machinery. As described by Typas et al. (2012), this may involve stretching of the peptidoglycan network hence influencing the activity of outer membrane-anchored lipoproteins. These proteins reach through the pores of the peptidoglycan network to interact with peptidoglycan synthases (penicillin binding proteins) as required for constriction (Woldringh et al., 1987). Proteins interfering with FtsZ-ring formation were recently also related to the NO phenomenon (reviewed by Wu and Errington, 2012).

The notion that a functional relationship exists between DNA dynamics and peptidoglycan biosynthesis is supported by the high correlations found between cell dimensions and the amount of DNA per nucleoid (*G*/*terC*) over a wide range of conditions (Zaritsky, 2015). Moreover, the constant aspect ratio (cell length/width ratio) supports the view that the expansion of the nucleoid during replication and segregation (and cell mass growth) occurs equally in three dimensions.

## Concluding Remarks

It is well known that the formulas describing cell mass and DNA content, as well as nucleoid complexity (amount of DNA per nucleoid), can only be applied in populations that grow under steady-state conditions (Campbell, 1957; Fishov et al., 1995). However, confirmation of steady state is seldom mentioned or documented. In many studies, bacterial batch cultures growing in rich media are used after a 100- to 1000-fold dilution of an overnight culture. In such populations the steady state has probably not been reached as it requires unperturbed, exponential growth at the same rate for some 20 generations (e.g., Maalϕe and Kjeldgaard, 1966).

How do single-cell growth studies in microfluidic channels measure up to the requirements for steady state growth? It appears that constancy of growth rate and length distributions of newborn cells dividing in the channels can accurately be monitored (Wang et al., 2010; Campos et al., 2014; Osella et al., 2014; Taheri-Araghi et al., 2015). If in addition the growth experiments could include observations on nucleoid extension and segregation after labeling with, for instance, fluorescent DNA binding proteins (e.g., Männik et al., 2012; Pelletier et al., 2012), it would be possible to test the present proposal, that DNA replication and cell growth are coupled via a segregation signal for cell division. If the presumed segregation signal could be related to forces exerted by the nucleoid on the plasma membrane (Rabinovitch et al., 2003) and on the peptidoglycan network (Typas et al., 2012), it would support a belief expressed by Bob Pritchard more than 50 years ago: “...that an understanding of the determination of cell size and shape will not be possible without taking into account the physical forces to which the cell boundary is exposed.” (Pritchard, 1974). We believe that the task of Physicists in expanding and deepening understanding of Cell Biology, bacteria included of course, is as critical as it was for Molecular Biology during the last Century, and similar, tight cooperation with Biologists is as crucial. The novel technologies continuously developed to enhance this end, as exemplified in the whole series of articles of this *Research Topic*, facilitate the study on both levels, single cells and single molecules in real-time.

In this memoir-style review, we try to bridge between past achievements and future prospects in the relatively-young field of Bacterial Physiology through present knowledge; scientists and students who are involved can exploit the information, which by no means is exhaustive, for the benefit of their current investigations, in the never-ending endeavor to understand Nature.

### Conflict of Interest Statement

The authors declare that the research was conducted in the absence of any commercial or financial relationships that could be construed as a potential conflict of interest.

## Footnotes

1 http://www2.le.ac.uk/news/blog/2014-archive-1/october/50th-anniversary-of-department-of-genetics-celebrates-world-changing-research-1

2 http://ariehz.weebly.com/uploads/2/9/6/1/29618953/emboworkshop1984.pdf
